# The Use and Perceived Usefulness of an Online Toolbox Targeted at Employers (MiLES Intervention) for Enhancing Successful Return to Work of Cancer Survivors

**DOI:** 10.1007/s10926-020-09929-4

**Published:** 2020-10-22

**Authors:** M. A. Greidanus, A. E. de Rijk, M. H. W. Frings-Dresen, C. M. Tiedtke, S. Brouwers, A. G. E. M. de Boer, S. J. Tamminga

**Affiliations:** 1 Department Public and Occupational Health/Coronel Institute of Occupational Health, Amsterdam Public Health Research Institute, Amsterdam UMC, University of Amsterdam, Meibergdreef 9, Amsterdam, The Netherlands; 2 Department of Social Medicine, Faculty of Health, Medicine and Life Sciences, Research Institute Primary Care and Public Health (CAPHRI), Maastricht University, Duboisdomein 30, Maastricht, The Netherlands; 3 Department of Public Health and Primary Care, Centre for Environment & Health, KU Leuven, Kapucijnenvoer 35, Leuven, Belgium

**Keywords:** Cancer survivors, Employment, Internet-based intervention, Return to work, Sick leave

## Abstract

**Purpose:**

The MiLES intervention is a web-based intervention targeted at employers with the objective of enhancing successful return to work (RTW) of cancer survivors. The aim of this study is to gain insight into the employers’ use and perceived usefulness of the MiLES intervention.

**Methods:**

Employer representatives (e.g. Human Resource managers and supervisors) were given access to the MiLES intervention, which contains, among others, interactive videos, conversation checklists and tailored tips. After six weeks, an online questionnaire gathered data on employers’ use and the perceived usefulness of the intervention. In-depth qualitative data on these topics were gathered during semi-structured interviews, which were analyzed using a content analysis.

**Results:**

Thirty-one eligible employers were included. Twenty-two of them filled out the questionnaire and twenty were interviewed. Typically, employers used the intervention 2–3 times, for 26 min per visit. The usefulness of the intervention scored 7.6 out of 10 points, and all employers would recommend it to colleagues. Employers’ use decreased when support needs were low and when the intervention did not correspond with their specific situation (e.g. complex reintegration trajectories). Employers perceived the intervention to be supporting and practically oriented. They appreciated the fact that the intervention was web-based and combined visual and textual content. The possibility of consulting specialized services for complex situations would further enhance its usefulness.

**Conclusion:**

The MiLES intervention provides employers with a useful tool in their daily practice. Its effectiveness for enhancing employers’ managerial skills and cancer survivors’ successful RTW is subject for further research.

## Background

Considering that almost half of all persons newly diagnosed with cancer are 15–67 years of age, and that the worldwide life expectancy after cancer is increasing, facilitating work participation of cancer survivors is paramount [1–3]. It is vitally important for the cancer survivors themselves, as work may contribute to the survivor’s financial security, social belonging and quality of life, but also for other stakeholders involved, such as employers and co-workers, and for society at large [4–8].

Many qualitative and quantitative studies, conducted in various countries with different legislative and insurance systems, substantiate the fact that the employer is an important stakeholder in enabling the employed cancer survivor to return to work [9, 10]. The employer is in a position to create appropriate preconditions for the cancer survivor to return to work by, among others, planning the return to work (RTW), supporting the cancer survivor practically and showing commitment [9]. However, employers have indicated that they are currently inadequately equipped or supported to fulfil their important role during the RTW of cancer survivors [6, 11, 12]. It is therefore imperative to develop and evaluate supporting tools to assist employers to provide adequate employer support for cancer survivors, in order to enhance cancer survivors’ RTW [7, 9, 11, 13–17]. Such tools do exist, both within organizations and by external specialized organizations, but these tools are primarily developed on the basis of practice, not on the basis of scientific evidence, and scientific evaluation is often not addressed [18, 19].

For this reason we developed the MiLES (“the Missing Link: optimizing the return to work of Employees diagnosed with cancer, by Supporting employers”) intervention targeting the employer during RTW of employees diagnosed with cancer [20]. The intervention endeavors to change the behavior of the employer. More specifically, the aim of the intervention is getting employers to perform the most important employer actions, such as providing emotional and practical support, communicating with the cancer survivor and planning the RTW [21], and thereby aims to optimize successful RTW of cancer survivors [20]. The intervention has been developed using the Intervention Mapping approach [22], and the trans-theoretical model of change was used as a theoretical framework [23]. The MiLES intervention is fully web-based, which enables employers to access the intervention when and where they prefer [20, 24], and thereby facilitates a periodic refresher if deemed necessary. The latter might be important, since face-to-face interventions targeting employer practices in regard to employees with non-cancer-related health problems, typically found positive short-term effects at employer level, such as improved knowledge and behavior, but effects on the longer term were either not measured or disappeared for most outcomes [25–28].

In addition to evaluating the effectiveness of the MiLES intervention for the cancer survivor, it is also vital to get insight into the use and perceived usefulness of the intervention among employers [29]. This is particularly important since a previous study indicated that involving employers in a cancer-related RTW intervention is challenging, and since knowledge on the use and usefulness of web-based interventions targeted at employers is lacking [30]. Knowledge and understanding of the employers’ use and perceived usefulness of the MiLES intervention can strengthen its uptake and impact in actual practice [29].

The following research questions were formulated:

1(a) How do employers evaluate their *use of the MiLES intervention* during the sickness absence and RTW of a cancer survivor, and (b) What barriers and facilitators for use are experienced by them?

2(a) How do employers perceive the *usefulness of the MiLES intervention* during the sickness absence and RTW of a cancer survivor, and (b) What rationales for their perceived usefulness do they provide?

## Methods

### Study Design

Employers were given access to the MiLES intervention for a period of six weeks, followed by an online questionnaire to gather quantitative data (*research questions 1a and 2a*) and a semi-structured interview to gather in-depth qualitative data (*research questions 1b and 2b*). The Medical Ethics Committee of the Academic Medical Center (AMC) had no objection to the conduct of this study, since participants are not subjected to compulsory procedures and their psychological condition is not in question (Reference Number W19_010 # 19.028). Informed consent was obtained from all individual participants included in the study. The STROBE checklist for cross-sectional studies was used to enhance accuracy and completeness of the reporting in this article [31].

### Participants: Employers

The inclusion criteria were: representing the employer, e.g. Human Resource (HR) manager or direct supervisor; being responsible for the work-related support of at least one employee with cancer at time of the study; being able to understand, speak and read Dutch sufficiently to participate. Recruitment took place until 41 employers were included. This sample size was based on a previous study with a similar aim and design that included 35 participants [32], supplemented with an expected 15–20% loss to follow-up. These numbers are also expected to safeguard data saturation on the basis of a previous qualitative study with employers [6].

### MiLES Intervention

The MiLES intervention is an open-access, web-based intervention. The overarching aim of the intervention is to optimize the successful RTW of cancer survivors, by supporting the employer. The trans-theoretical model of change was used as theoretical framework, as it contributes to the understanding of behavior change and guided the decision on appropriate methodologies and practical strategies to induce the targeted behavior change [20, 23]. The intervention consists of textual content, visual content (i.e. an animation and interactive communication videos), links to external sources (i.e. blogs and videos on the cancer survivor’s perspective, and external websites concerning privacy, external expertise, and laws and regulations), and practical tools (i.e. conversation checklists). The intervention targets the employer, and stimulates the employer to take the most important employer actions, such as to communicate, provide support, assess the cancer survivor’s work ability and show appreciation [21]. Its content is tailored per RTW phase, as the following phases are distinguished: (1) *disclosure*, (2) *treatment*, (3) *RTW planning*, and (4) *actual RTW*. The intervention distinguishes three “experience types” of cancer survivors, on the basis of their experience with their work disability due to cancer: (1) an *emotional cancer survivor*, in which intense emotions such as sadness and anger can alternate quickly; (2) a *cancer survivor who wants little attention for their health situation*, and wants to be involved in work for as long as possible and return to work as quickly as possible, and; (3) a *cancer survivor who starts looking differently at work and life*, and gives other priorities due to their illness [33]. A comprehensive description of the development, design and content of the MiLES intervention has been published elsewhere [20].

### Procedures

Employers were recruited from January 2019 through July 2019, via oncological occupational physicians, social media, relevant platforms, and websites for employers and HR personnel, and through snowball sampling. Employers signed informed consent forms directly via the Qualtrics online questionnaire system (https://www.qualtrics.com), a move designed to lower the threshold for participation in the study. Employers could contact the researchers (MG or SB) at any time by telephone before deciding whether to participate.

After inclusion, the employers received the URL giving them unlimited access to the MiLES intervention for a period of 6 weeks. Considering Dutch legislation, which obligates employers to discuss the progress of RTW with their employee on sick-leave every 6 weeks [34], this period would entail at least one meeting between the participating employer and the cancer survivor employed by them. As we expected that participating employers would be able to assess the usefulness of the MiLES intervention after one such meeting, a follow-up period of six weeks was expected to be appropriate. The use of the intervention was not mandatory for participating employers, but completely voluntary. Six weeks after registration, the participant received an invitation to fill out an online questionnaire and participate in a telephone interview. To make sure all participants met the inclusion criteria, all inclusion criteria were checked during the interview and participants who did not meet the criteria were retroactively excluded. In the event that a participant dropped out before the telephone interview, the participant was asked by e-mail whether he or she met the inclusion criteria. Quantitative data (see below) from these participants were only incorporated in the event of an affirmative answer by e-mail.

### Data Collection

Qualtrics survey software (Qualtrics, Provo, UT, https://www.qualtrics.com) was used for the 10–15 min questionnaire, to gather quantitative data on the use and perceived usefulness of the MiLES intervention (*research questions 1a and 2a*), and to gather the participant characteristics. After the participant completed the questionnaire, a semi-structured telephone interview of ± 20 min was scheduled to gather in-depth qualitative data (*research questions 1b and 2b*). An interview guide was designed and the participant’s answers to the questionnaire served as input for the interview (see Table 1 for the topic list). The interviews were conducted by either MG or SB and consisted of open-ended questions to evoke comprehensive responses and gather a thorough understanding of the participants’ thoughts [35]. At the end of each interview, the interviewer summarized the main outcomes and asked the participant whether he or she would want to reflect to or add to this. The interviews were audio recorded and transcribed verbatim by MG or SB. The interviewers monitored and discussed data saturation after every 3–5 interviews, and discussed and decided whether to change the sequence of the topics addressed during the interview in order to get more time to address specific topics. Data saturation was reached when no new information on a specific topic was mentioned in three consecutive interviews.

**Table 1:** Topic list interviews

Topic	Sub topics
*Use of the MiLES intervention*	Use of the intervention in general Reasons (not) to use intervention When and where the intervention has been used Barriers to and facilitators for using the intervention (*e.g. personal factors, organizational factors or technical issues*)
*Usefulness of the MiLES intervention*	Usefulness of the intervention in general Rationale behind the perceived usefulness Usefulness of parts of the intervention: Animation homepage Tips per RTW phase Communication videos External blogs and videos concerning the cancer survivor’s perspective External websites concerning privacy, external expertise, and laws and regulations Conversation checklists Suggestions for improving the usefulness of the intervention
*Usefulness in respect of willingness to support*	Usefulness in respect of the employer’s willingness to support in general Usefulness in respect of the following “willingness-related” topics: Perception of the cancer survivor Perception of most important employer actions Suggestions for improving the usefulness of the intervention in respect of increasing the employer’s willingness to support
*Usefulness in respect of ability to support*	Usefulness in respect of the employer’s ability to support in general Usefulness in respect of the following “ability-related” topics: Knowledge to support cancer survivors Skills regarding supporting cancer survivors Ability to deal with external factors Suggestions for improving the usefulness of the intervention in respect of increasing the employer’s ability to support

All questions were individualized based on the quantitative answers of the participant. For each question, several alternative questions were asked, in order to get in-depth insights into the topics and sub topics concerned

#### Participant Characteristics

The following demographics and work-related characteristics were assessed in the online questionnaire: age (< 50; ≥ 50 years), gender, level of education (secondary education; intermediate vocational education; higher professional education), function (HR manager; direct supervisor; other), years of experience in current position, number of cancer survivors supervised in current position, organization size (≤ 50; 51–250; ≥ 251 employees), and sector to which the organization belongs (non-profit; profit; other). Participants were also asked to indicate in which RTW phase the cancer survivor was during the study [21], and to assess the “experience type” of this cancer survivor, on the basis of the above-mentioned predefined types [33].

#### Use of the MiLES Intervention

The participants’ self-reported use of the intervention, during the follow-up period, was assessed in the online questionnaire (*research question 1a*). The following data were collected: whether or not the participants used the intervention (yes; no), the number of visits, the average duration of the visits, and whether or not the participant used each part of the intervention (e.g. the communication videos, the tips for each RTW phase and the communication checklists). During the interviews, participants were asked which barriers to and facilitators for the use of the intervention they experienced (*research question 1b*) (Table 1).

#### Usefulness of the MiLES Intervention

The online questionnaire included the following items relating to the usefulness of the intervention (*research question 2a*): usefulness in general (1–10 scale, higher score indicating more useful), whether they would recommend the intervention to a colleague (yes; no), usefulness of the intervention in its entirety and each part of the intervention separately (e.g. the communication videos, the tips for each RTW phase and the communication checklists) (useful; somewhat useful; not useful), and whether the intervention increased their willingness and ability to support the cancer survivor (increased; somewhat increased; not increased) [9]. Items were only displayed when the participant indicated that he or she used (the corresponding part of) the intervention.

During the interviews, open-ended questions were asked in the event the participant rated a certain part of the intervention either useful or not useful (*research question 2b*). In order to reduce interviewee burden, neutral answers in the online questionnaires, i.e. “somewhat useful”, were not explicitly addressed. Participants were also asked whether they had suggestions for improving the usefulness of the intervention (Table 1).

### Data Analysis

Descriptive statistics (SPSS version 25.0; IBM Corp., Armonk, NY) was used to analyze participant characteristics and quantitative data, and to determine whether participant characteristics differed between participants that used the intervention (from now on: “users”) and the ones that did not (from now on: “non-users”).

Interviews were analyzed using MAXQDA software (Verbi software GmbH, Marburg 2007). MG performed a directed content analysis [36], composed of: 1) distributing and coding parts of the interviews into the topics and subtopics given in the interview guide (codes represented the text as closely as possible); 2) clustering codes on a relatively low level of abstraction (for each topic and subtopic separately), resulting in clusters such as “visual content is useful” and “textual tips are practical”; and 3) identifying and defining themes per topic and subtopic. For four interviews, the two richest interviews (i.e. those with most codes assigned by MG) and two random interviews of the employers grading the usefulness of the intervention with the highest and the lowest score, CT repeated all the steps of the analysis blindly (i.e. without any information on earlier analysis). Thereafter, CT and MG discussed and compared the codes and themes that emanated from their analyses, and checked whether the codes and themes CT identified on the basis of the four interviews were incorporated in the codes and themes MG identified on the basis of all interviews. Any missing codes and themes were discussed in the light of the original interview, after which it was decided whether to add these to the final codes and themes. The analyses of MG and CT with regard to these four interviews showed great similarities, and double blind analysis of all interviews was therefore deemed not necessary.

## Results

**Fig. 1:**
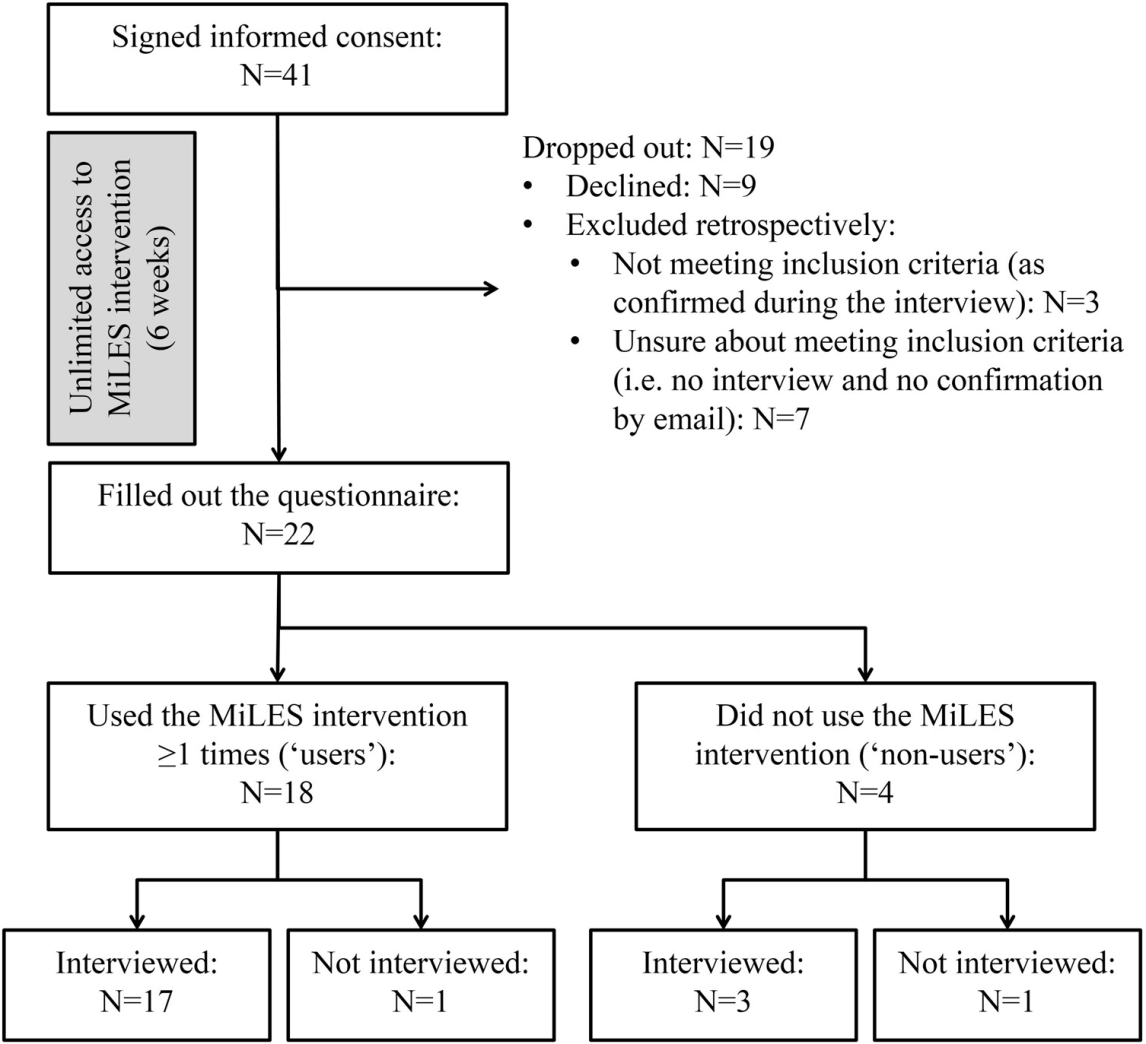
Participant flow chart. N = sample size

Forty-one employers signed informed consent forms, of which ten were retrospectively excluded, given that they either did not meet the inclusion criteria (N = 3) or that it was unclear whether or not they met the inclusion criteria (N = 7) (Fig. 1). Of the other 31 participants, nine (29%) dropped out as they had not filled out the questionnaire. Ultimately, 22 employers filled out the questionnaire, of which 20 were interviewed for, on average, 14 min (SD = 5) (two employers declined the interview for unknown reasons). Data saturation was reached for the qualitative data on both the use (*research question 1b*) and the usefulness (*research question 2b*) of the intervention, except for the usefulness of the *links to external sources* and the usefulness to increase participants’ *willingness* and *ability to support*. The participant group comprised an equal number of men and women (N = 11; 50%), mostly working at large-sized organizations (N = 15; 68%) and in the non-profit sector (N = 16; 73%), and the group mostly consisted of direct supervisors (N = 15; 68%) (Table 2).

**Table 2:** Participant characteristics

Participant characteristics (N = 22)		
	*N (%)*	*Mean (range)*
Age		
< 50 years	11 (50)	
Gender		
Male	11 (50)	
Level of education		
Secondary education Intermediate vocational education Higher professional education	2 (9) 14 (64) 6 (27)	
Size of organization		
< 50 employees 51–250 employees > 251 employee	2 (9) 5 (23) 15 (68)	
Sector to which organization belongs		
Non-profit Profit	16 (73) 6 (27)	
Position		
HR manager Direct supervisor Re-integration coach	6 (27) 15 (68) 1 (5)	
Experience in current position		
Years Number of cancer survivors		11 (1–30) 3 (1–10)
RTW phase of cancer survivor [21]		
Phase 1: disclosure Phase 2: treatment Phase 3: RTW planning Phase 4: actual RTW	2 (9) 10 (46) 3 (14) 7 (32)	
“Experience type” of cancer survivor [33]		
An emotional cancer survivor A cancer survivor who wants little attention for their health situation A cancer survivor who starts looking differently at work and life “I cannot judge”	4 (18) 11 (50) 7 (32) 0 (0)	

*N* sample size, *HR* Human Resource, *RTW* return to work

### Use of the MiLES Intervention

*Research question 1a*: Eighteen of the 22 participants (82%) that filled out the questionnaire used the intervention at least once (i.e. users). The users used the intervention on average 2.4 times and 26 min per visit (Table 3). Among the users, the most frequently used parts of the intervention were the tips per RTW phase (N = 17; 94%), the animation (N = 16; 67%) and the conversation checklists (N = 10; 56%). The users and non-users did not differ as to the measured demographics and work-related characteristics (data not shown).

**Table 3:**
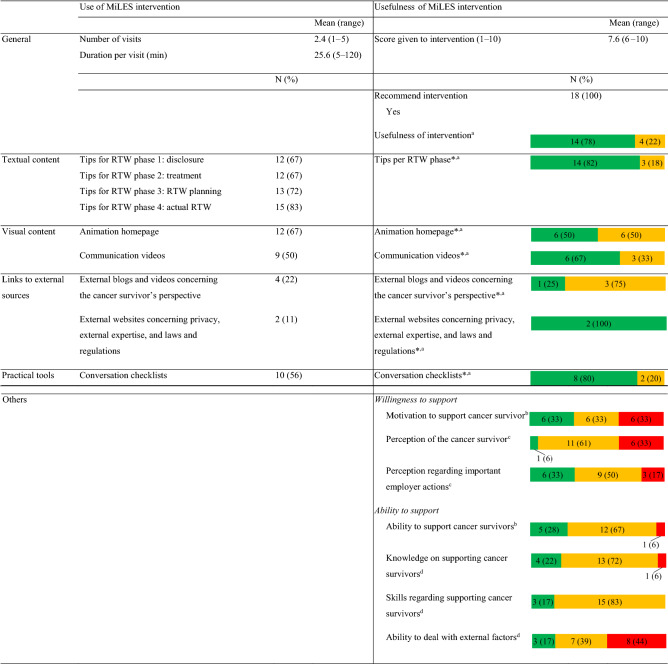
Results of the questionnaire on the use and perceived usefulness of the MiLES intervention, filled out by the “users” (N = 18)

*SD* standard deviation, *N* sample size, *RTW* return to work, *min* minutes

^a^Answer categories: Useful (green), somewhat useful (orange), not useful (red)

^b^Answer categories: Increased (green), somewhat increased (orange), not increased (red)

^c^Answer categories: Changed (green), somewhat changed (orange), not changed (red)

^d^Answer categories: Improved (green), somewhat improved (orange), not improved (red)

^*^This question was only filled out by participants that used the relevant part of the MiLES intervention

*Research question 1b*: During the interviews, participants indicated that they used the intervention mostly at work, or possibly at a place where nobody would disturb or watch them, just before the conversation with the cancer survivor:

I really studied it beforehand, before the conversation and used it to determine the tactical tone of the conversation (…) to prepare the conversation and to formulate certain questions in my head. So I would not be at a loss of words. [direct supervisor].

Participants were motivated to use the intervention by intrinsic as well as extrinsic factors. As to the intrinsic facilitators, participants indicated that they felt responsible and were motivated to provide the best possible support to the cancer survivor:

I’m just very intrinsically motivated to support colleagues, in any form whatsoever (…) and if you are diagnosed with cancer (…) that simply does a lot with somebody. I really want as many tools as possible (…) to get someone through that… [direct supervisor].

As to the extrinsic factors, some participants indicated that their occupational physician or HR department drew attention to the study and the intervention, thus serving as a facilitator for these participants to use the intervention. Other facilitators for the use of the intervention where the fact that the structure and layout were clear and the intervention was easily accessible and that the intervention aligns with the employers’ daily practice. Participants mentioned that their use of the intervention would further increase once the statutory steps required were better integrated in the intervention.

Some interviewed non-users mentioned that, despite having registered for the study, they were not aware of the existence of the intervention, or that the e-mail with the URL of the intervention escaped their attention in the hustle and bustle of the day:

…you sign up, and I undoubtedly received it [the URL, resp.] proper, but I get a lot of emails in a week, and I think it escaped my attention… [direct supervisor].

A few users also perceived some barriers for using the intervention. For them, the specific situation of the cancer survivor was not addressed in the intervention, for example when the RTW phases that structure the intervention did not apply to the cancer survivor’s situation:

…planning RTW and actual RTW [RTW phases 3 and 4, resp.] are somewhat arbitrary (…) because you suggest that, during treatment, people are primarily focused on the treatment. (…) But [in my specific case] there was no question of a return to work, since the contact always remained. [direct supervisor].

The use of the intervention also depended on the difficulty of the specific situation with the cancer survivor, since some participants simply needed very little support in guiding the cancer survivor, which fact was perceived as a barrier to their use of the intervention:

…so that's why at the moment I also spend a little less time (…) on your website (…), because, it sounds a bit, I don't mean that, (…) [but the cancer survivor is] a somewhat easier case. [direct supervisor].

Lastly, some IT-related barriers were perceived, for example that the IT system at work blocked the intervention, that the conversation videos lagged and that some parts of the intervention were not visible on smaller screens.

### ***Usefulness of the MiLES ***Intervention

*Research question 2a*: Participants who used the intervention gave the usefulness of the intervention a score of 7.6 out of 10 points, and all indicated that they would recommend the intervention to a colleague (Table 3). None of the participants indicated that they found the intervention or any of the parts of the intervention “not useful”. Of the parts of the intervention that have been used by at least nine participants, most participants perceived the textual tips per RTW phase (N = 14; 82%), the conversation checklists (N = 8; 80%) and the communication videos (N = 6; 67%) to be “useful”. Lastly, 94% (N = 17) of the participants indicated that the intervention at least “somewhat increased” their ability to support, and 66% (N = 12) indicated that the intervention at least “somewhat increased” their motivation to support cancer survivors.

*Research question 2b*: In general, the interviewed participants indicated that they perceived the intervention as supporting and useful at times when they experienced uncertainty during the sickness absence and RTW of the cancer survivor. Participants indicated that they considered the content of the intervention to be complete, that it was practically oriented and clear, and that the combination between the visual and the textual content appealed to them:

…of course, everyone is triggered by something else. There are more visually oriented persons and persons who [prefer to read]. So I think that by offering both, one can have the choice to see what appeals to them. [direct supervisor].

Some participants recognized their own actions in the content of the intervention, and mentioned that the intervention was especially useful for unexperienced employers or employers who only occasionally encounter cancer survivors. For themselves, the intervention was a welcome confirmation of their own good employment practices:

Well, it was more of a positive confirmation. It is always nice to get confirmation that what you did was the right approach. (…) So in that sense it was useful to me. [HR manager].

The intervention was perceived as less useful when the situations outlined did not match their current practice or the hierarchy between participant and cancer survivor, or when the situations were unrealistically positive:

The assumption that everything [all conversations, resp.] takes place in the office… you know, the reality is different. [HR manager].

A few additions to the intervention were suggested by participants, to enhance its usefulness or usability: a clear introduction or overview on the homepage, general information about cancer and national legislations, suggestions for the allocation of tasks within the organization (e.g. between the HR manager, direct supervisor and occupational physician), and a forum offering the possibility to consult with fellow employers. Lastly, participants suggested that the intervention should include the possibility to engage a specialized coach for situations that are more complex:

… maybe there should be something else for that too, when it gets complicated, that as a manager you can talk to a coach who is specialized in that. (…) [This intervention] has to offer 80%, and the rest has to be done in another way. [direct supervisor].

The *textual content* of the intervention was practically oriented, written with a good information density, and provided guidance throughout the different RTW phases. The phases themselves were clear and helped to put the situation being dealt with into perspective. Nevertheless, the phases were not adequate for all situations, and the participants suggested adding a 5^th^ phase to the intervention:

…the question is whether [this cancer survivor] will survive. So, those are really tough things. Then it does not make sense to say, well, how are we going to plan your return to work. But you are still the manager and it is your task to, let’s say, guide this in a different way, and I miss that a little in the [intervention]. [direct supervisor].

The *visual content* was perceived to be useful by being illustrative and insightful as to the importance of listening carefully to the cancer survivor, and to the realization that there are different types of cancer survivors:

… what I think is good, is that it is clearly said, well, cancer survivors can be different, and that is okay, but it is good to realize that and to know that you have to react and act differently. [HR manager].

Some participants indicated that they missed a particular type of cancer survivor, for example a non-cooperative cancer survivor who wants to slow down the RTW or an even more emotional cancer survivor. However, the three types were recognized and found practicable by the participants:

The videos also give insight into some form of interviewing, because they separate [different types of cancer survivors]. We see all these persons in our own organization. So when one of our employees is diagnosed with cancer, one can always apply one of these three types, I think. [re-integration coach].

Participants mentioned that the conversation checklists provides structure, overview and guidance, and prevented participants from forgetting subjects in the emotional setting of such a conversation:

I found these quite difficult and emotional conversations. (…) [the conversation checklist] is a good guide to have with you. So that you do not skip steps and do not forget to ask certain things (…), like: ‘We have now discussed this and this and agreed this and this…’. So it was very reassuring for me to work with [HR manager].

The intervention also had a motivational effect, for example to start an open dialogue with the cancer survivor:

… that was really a question that played in my head: should I ask about it or shouldn’t I ask about the situation, because then you feel like an “intruder”. So then you think: well, I should not ask about it. But the [intervention] is very clear that you have to keep asking (…): how are you doing, what is your status, what is on your mind? That was really an eye-opener to me, and it was really the most important lesson I’ve learned. [direct supervisor].

Others mentioned that their motivation had not been much increased, as they had already been highly motivated to support the cancer survivor:

My motivation to support this employee was great from the start anyway (…). This tool has only helped me to determine the implementation of the support, but not the motivation in itself increased. [direct supervisor].

Participants mentioned that the intervention increased their knowledge and awareness of how cancer survivors may experience their sickness, and how to act or what to ask. This knowledge was perceived to be closely linked to their skills, as participants perceived both their communication skills and skills at being understanding to have increased:

I think that if you have more knowledge, your skills will always get better. (…) watching the movies and reading the information increases your knowledge, and that helps to enter such a conversation better. And with that, your skills are also increased. [direct supervisor].

Lastly, for some participants, the intervention did not increase their knowledge, but served as a confirmation of what they already knew.

## Discussion

### Main Findings

This study aimed to get insight into the employers’ use and the perceived utility of the MiLES intervention in a survey and interview study among 31 employers approached with convenience sampling. Most employers used the intervention – typically 2–3 times, for an average of 26 min per visit. The participants who were interviewed described the intervention as helpful and providing support when they experienced uncertainty, and affirmative of their good employment practices; its practical relevance and its combination of visual content, textual content and practical tools appealed them.

### Comparison with the Literature

We found that employers included in this study valued the fact that the intervention was web-based, since this enabled them to access its content when and where they preferred, for example at work right before a meeting with the cancer survivor. In contrast, a systematic review of ten studies with interventions aiming to enhance employer practices concerning employees with mental health problems shows that these interventions were predominantly face-to-face [27]. These interventions consisted of a 2–14 h training program covering mental health knowledge and awareness, promoting a positive workplace, and developing skills to best support and best react to employees’ mental health issues [27]. Such face-to-face interventions have evidently some advantages compared to online materials, such as the possibility to interact and discuss with fellow employers and to consult more specialized support for complex situations, which was also requested by several participating employers. However, as found in the current study, offering an intervention via the Internet facilitates appropriate timing of the intervention, makes the intervention easily accessible and presumably less time consuming, and facilitates the implementation of a periodic refresher of its content if needed. The latter may be relevant to sustain positive effects on employer practices [25–27]. Taking into consideration the above-mentioned, we recommend future interventions targeting employers during the RTW of cancer survivors to provide predominantly online materials, supplemented with the option to consult specialized services when needed, making them easily assessable for employers and enabling repeated and timely exposure.

Previous studies have shown the significance for employers of collaborating with other stakeholders in the RTW of cancer survivors, e.g. colleagues and occupational physicians, and the needs of employers to be supported in order to strengthen this collaboration [6, 9, 12, 37–39]. The intervention was thought to meet these needs, since it included links to oncological occupational physicians and references to possible specialized re-integration services. These links appeared inadequate to meeting the concerning needs of employers; this fact is reflected in the relatively low scores for the usefulness to increase the employers’ ability to deal with external factors, and the mentioned need for additional specialized support in the case of complex situations. According to the employers, the intervention is primarily focused on the interaction with and managing of the cancer survivor, rather than the interaction with other stakeholders. We therefore recommend the intervention to provide more insight into the different roles and responsibilities of actors in the RTW of cancer survivors, such as HR managers and occupational physicians, and to support employers to collaborate with these actors. In addition, we recommend that the network of possible specialized services, among others with experiencers, be expanded and that it be made easier for employers to reach out to these services when needed [24].

### Strengths and Limitations

The strength of the current study is the diversity of the sample, including perspectives of both HR managers and direct supervisors, who were both positive and critical and who had different experiences with cancer – which enhances the generalizability of the results. Another strength is that both quantitative and qualitative data were gathered, providing profound insight into the use and perceived usefulness of the MiLES intervention. Such an evaluation and understanding of the process of the intervention is an important step, opening the black box of the intervention instead of only evaluating its effect at the level of the cancer survivor [29].

Limitations are, firstly, that non-profit and larger organizations were overrepresented among the study sample. Since RTW trajectories and experiences are dependent on the work environment concerned [9], the generalizability of the perceptions found to smaller organizations or competitive, profit organizations is unclear. In smaller organizations employers assumedly have less experience with cancer survivors at work, and might run into specific challenges, such as limited possibilities for work adjustments, more intense emotional impact for colleagues, less access to and financial resources for supporting services, and more far-reaching financial consequences of long-term sickness absence [24, 40, 41]. One might expect that these specific challenges would enhance the need for and usefulness of supportive interventions such as the MiLES intervention. Secondly, we did not measure characteristics of employers who dropped out before filling out the online questionnaire, nor recorded reasons for them to drop out of the study. It is therefore unclear whether characteristics of employers who dropped out differ from the ones that did not drop out, and whether the current study reflects perspectives of employers who were positive about the intervention [42].

### Implications and Recommendations for Future Research and Practice

In addition to the above-mentioned recommendations to deliver future interventions targeted at employers predominantly with online materials, and to facilitate the collaboration with other actors in the RTW of cancer survivors, we recommend the following for future research and practice.

Firstly, for future studies among employers, we recommend the continued employment of measures to lower the burden for participation, and registration of data on the efficacy of the individual recruitment channels. Since a previous study has shown the difficulty of engaging employers in work-related interventions [30], various recruitment channels were employed in the current study. By lowering the threshold to participation by direct subscription for the study, we were able to include a sufficient number of employers in the study. However, a relatively high dropout rate could not be avoided. We tried to minimize selection bias towards positive and willing employers, which is a common problem in research among employers [6, 12, 37, 43], but a certain extent of selection bias cannot be ruled out. Registering data on the efficacy of the individual recruitment channels can provide valuable insight into employers’ motives for participating and on how to persuade them to participate.

Secondly, we recommend that future studies investigate the role and needs of employers in the event that an employed cancer survivor is not able ever to return to work due to worsening health or death. Previous studies and the current study have demonstrated that employers may struggle with their role in this situation, and need support to fulfil this role properly [6, 12].

Thirdly, future studies should reveal what should be an appropriate route for implementing work-related interventions targeting the employer in organizations. The current study endorses the importance of appropriate timing of the intervention, as employers only used the intervention when they experienced support needs relating to the situation with the cancer survivor. Future studies should therefore define what stakeholder could best be responsible (e.g. HR services, occupational physicians of external reintegration services), or what platform is suitable (e.g. a national website or organization intranet), to make sure that the intervention is provided as soon as the support needs of the employer arise.

Fourthly, we recommend for the development of future interventions targeted at employers to mobilize a participative approach and an adequate theoretical framework that underpins the intervention [22, 44]. This increases the likelihood that the intervention aligns with the specific needs of employers in practice, which was also recognized by employers who participated in this study, and may contribute to achieving the targeted behavior change [22, 44].

Lastly, this study reflects the perspectives of employers about the MiLES intervention. Although this is an important step that can strengthen the uptake and impact in actual practice, positive perspectives do not guarantee actual improved employer practices [29]. An effect study is thus important. Only then will it be possible to draw conclusion as to whether or not support targeted to employers can truly induce better work outcomes of persons diagnosed with cancer.
